# Supplementary material for: Past and recent anthropogenic pressures drive rapid changes in riverine fish communities
Source: Nat Ecol Evol. 2024 Jan 30;8(3):442–53. doi: 10.1038/s41559-023-02271-x (PMC10927561; doi:10.1038/s41559-023-02271-x)
Supplement: Supplementary file 1 — Supplementary Figs. 1–4 and Tables 1–5. [file 41559_2023_2271_MOESM1_ESM.pdf]

nature ecology & evolution

Article https://doi.org/10.1038/s41559-023-02271-x

Past and recent anthropogenic pressures drive rapid changes in riverine fish communities

In the format provided by the authors and unedited

Supplementary Figures

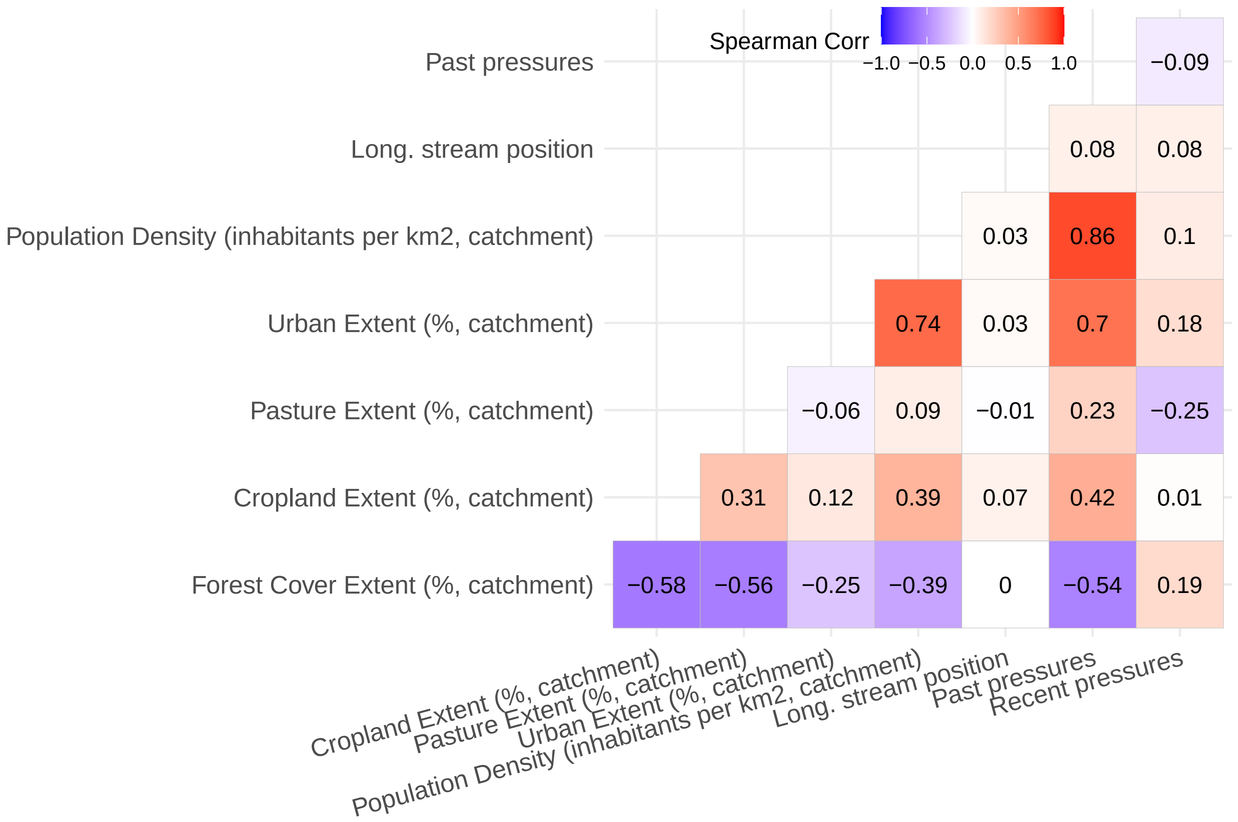

Supplementary Figure 1: **Spearman correlations among the ecological drivers** used in the hierarchical Bayesian model as well as with additional population density land use variables. Land use data and population density were extracted from RiverAtlas and are meant to illustrate the covariations of various human pressures with the human footprint index. We show the Spearman correlation with the extents of forest, pasture, and cropland for year 2000, urban extent for year 2015 and population density for year 2010 (See Linke et al., 2019 for details). We observe very low correlations among the ecological drivers (longitudinal stream position, past pressures and recent pressures). Past pressures (human footprint index for year 1993) are positively correlated with population density, urban extent and cropland extent, but are negatively correlated with forest extent.

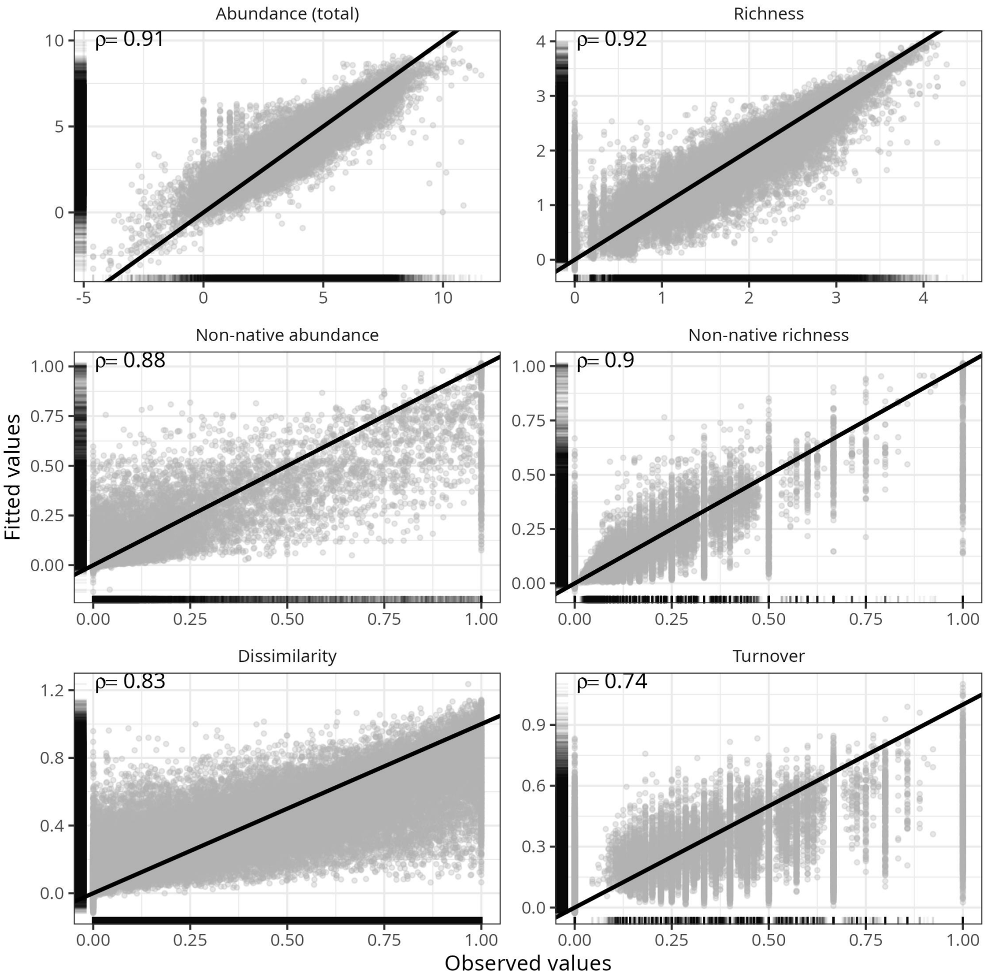

Supplementary Figure 2: **Fitted values of the models vs observed values** (N = 46932 sampling events). The black line displays the bisection, i.e. the intercept and slope are respectively 0 and 1. Sticks located at the abscissa and ordinate axis represent the density of values. The Pearson's correlation coefficient is displayed in each panel. Total abundance and species richness values are log-transformed. The model predicted the data reasonnably well, but it tended to overestimate low values and underestimate high values, especially for the proportion of non-native species and dissimilarity.

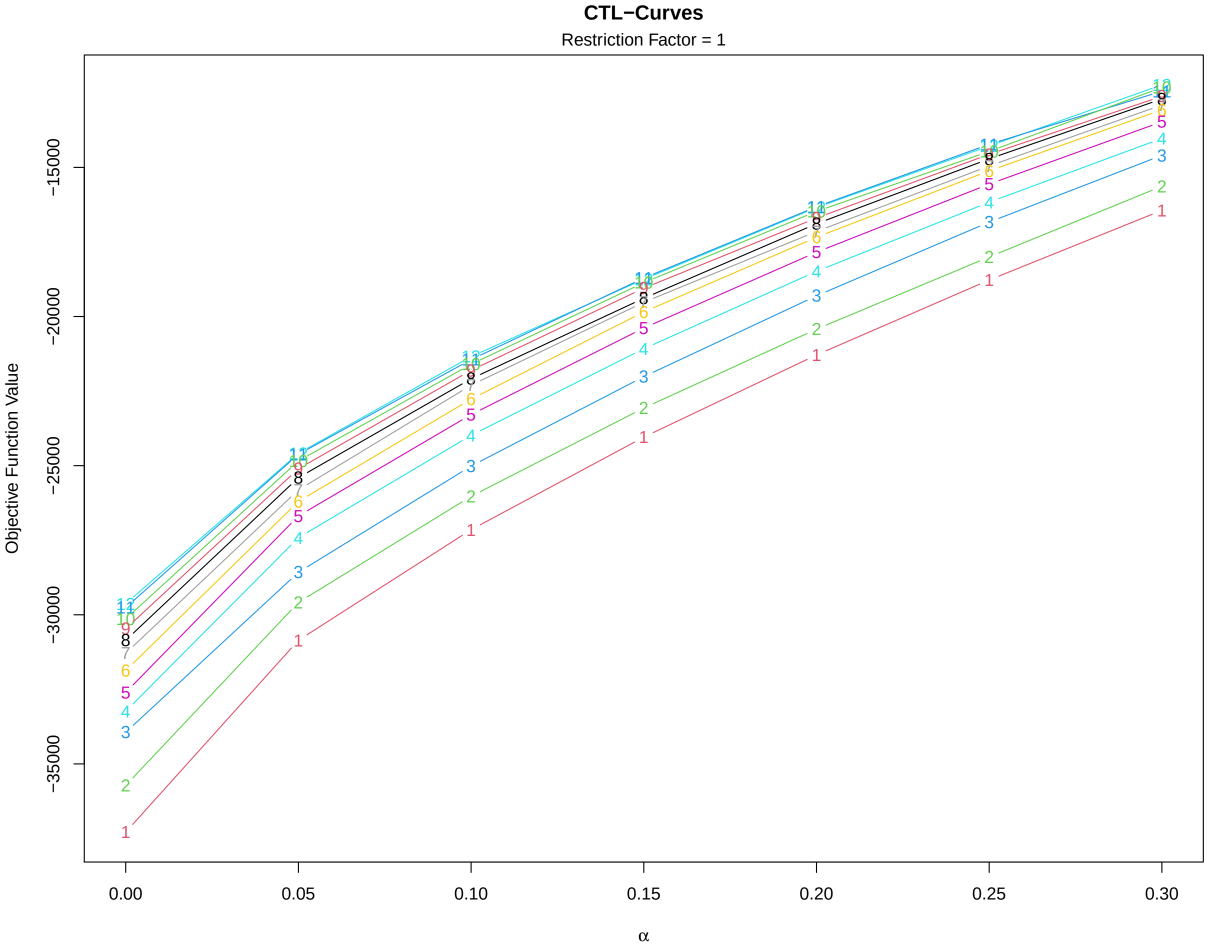

Supplementary Figure 3: **Objective function quantifying the goodness of the k-means clustering** according to the percentage of data being removed (the most outliers data in the multidimentional space). The different lines indicate differences in the goodness of fit for different number of clusters, as indicated by the numbers. In the results presented in the main text, we removed 5% of the data ($\alpha = 5\%$). We chose six clusters for our analyses as the goodness of fit increased only marginally from 6 to 7 clusters (and beyond). However, because of the small increases in goodness of fit observed from 4 to 5 clusters, we also tested the sensitivity of our conclusions regarding the community trajectories using 4 clusters (see Fig. S4).

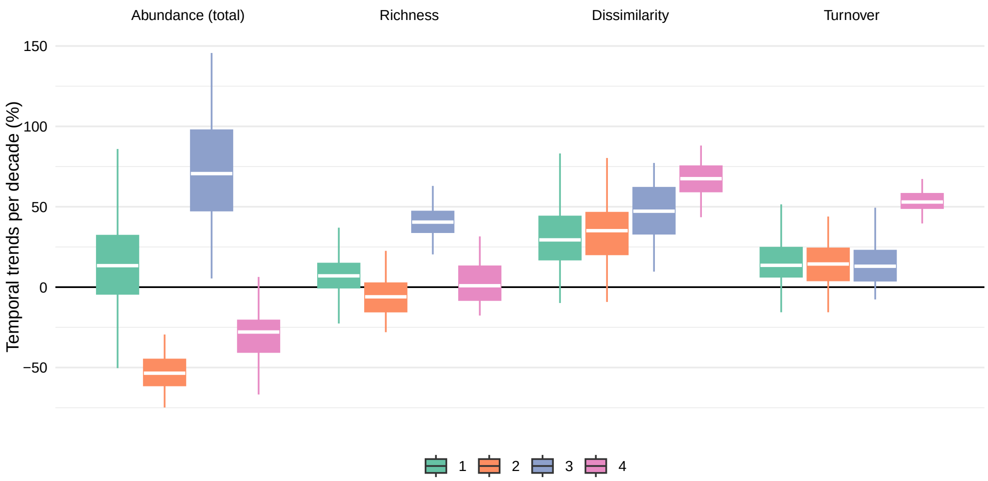

Supplementary Figure 4: **Distribution of temporal trends per decade by variable and cluster, with four clusters**. With four clusters instead of six, we do not observe the clusters related to changes in community composition (Fig. 2, main text). The center of the box depicts the median while the bounds depicts the 25% and 75% percentile. The whiskers depict the extreme values within 1.5 interquartile range beyond the bounds of the box.

Supplementary Tables

Supplementary Table 1: **References of the dataset included in the study**, arranged by sampling size. Numeric Source ID corresponds to the ID from the RivFishTIME database (Comte et al., 2021a). N: number of sites.

Source ID	Country	N	Reference
27	GBR	1284	U.K. Environmental Agency (2019) National Fish Populations Database (NFPD): Freshwater fish survey relational datasets. Available at https://data.gov.uk/dataset/d129b21c-9e59-4913-91d2-82faef1862dd/nfpd-freshwater-fish-survey-relational-datasets [Accessed on 10/15/2019].
32	FRA	1068	Office français de la biodiversité (2019) Suivi des éléments biologiques 'POISSONS' des rivières françaises. Available at http://www.naiades.eaufrance.fr/acces-donnees#/hydrobiologie [Accessed on 07/08/2019].
42	SWE	819	Sers, B. (2013) Swedish Electrofishing RegiSter – SERS. Swedish University of Agricultural Sciences (SLU), Department of Aquatic Resources. Available at http://www.slu.se/elfiskeregistret [Accessed on 11/18/2019].
MARIS	USA	227	U.S. Geological Survey, Core Science Analytics and Synthesis Program, 20131201, Multistate Aquatic Resources Information System (MARIS): United States Geological Survey, https://doi.org/10.5066/F7988525.
26	USA	79	U.S. Geological Survey (2019) BioData - Aquatic Bioassessment Data for the Nation. Available at https://apps.usgs.gov/biodata/ [Accessed on 25/05/2023], doi:10.5066/F77W698B.
Maryland	USA	73	Montgomery county monitoring program (2018). Available at https://www.montgomerycountymd.gov/water/streams/data.html
25	USA	55	Montana, Fish, Wildlife & Parks (2019) Fish Survey Sites. Available at http://gis-mtfwp.opendata.arcgis.com/datasets/8192e75218c6460ba97ba3dd0a2fb3a5 [Accessed on 05/21/2019].
21	USA	50	Long Term Resource Monitoring Program (2016) Available at https://www.umesc.usgs.gov/data_library/fisheries/fish1_query.shtml [Accessed on 06/09/2019].
28	USA	42	North Carolina Department of Environmental Quality (2018) Fish Community Assessment Data. Available at https://deq.nc.gov/about/divisions/water-resources/water-resources-data/water-sciences-home-page/biological-assessment-branch/fish-community [Accessed on 02/06/2018].
Ohio	USA	40	Ohio statewide monitoring program (2018). Available at https://www.orsanco.org/programs/fish-population/
15	USA	40	Iowa DNR [Department of Natural Resources] (2013) BioNet - Iowa DNR Biological Monitoring and Assessment Database. Available at https://programs.iowadnr.gov/bionet/ [Accessed on 02/01/2018].
20	USA	28	McLarney, W.O., Meador, J. & Chamblee, J. (2013) Upper Little Tennessee River biomonitoring program database. Coweeta Long Term Ecological Research Program. Available at: https://coweeta.uga.edu/dbpublic/dataset_details.asp?accession54045 [Accessed on 11/15/2019].
24	USA	22	Minnesota Pollution Control Agency (2018) Surface water data. Available at https://webapp.pca.state.mn.us/surface-water [Accessed on 04/11/2023].

(Continued on Next Page...)

Supplementary Table 1: **References of the dataset included in the study**, arranged by sampling size. Numeric Source ID corresponds to the ID from the RivFishTIME database (Comte et al., 2021a). N: number of sites. *(continued)*

Source ID	Country	N	Reference
30	USA	20	Winston, M.R., Taylor, C.M. & Pigg, J. (1991) Upstream extirpation of four minnow species due to damming of a prairie stream. Transactions of the American Fisheries Society, 120, 98–105. Taylor, C.M. (2010) Covariation among plains stream fish assemblages, flow regimes, and patterns of water use. In: Community ecology of stream fishes: concepts, approaches, and techniques. American Fisheries Society Symposium, 2010.;
36	USA	15	Davenport, S. R. (Unpublished data).
5	USA	8	Gammon, J. (2003) The fish communities of Big Raccoon Creek 1981-2002. Report for Heritage Environmental Services. Indianapolis, Indiana. 258 pages.
41	USA	7	Stefferud, J.A. (Unpublished data).
45	USA	7	Rinne, J.N., & Miller, D. (2006) Hydrology, geomorphology and management: implications for sustainability of native southwestern fishes. Reviews in Fisheries Sciences 14, 91–110.
10	USA	6	Gido, K.B, Propst, D.L., Olden, J.D., & Bestgen, K.R. (2013) Multidecadal responses of native and introduced fishes to natural and altered flow regimes in the American Southwest. Canadian Journal Fisheries and Aquatic Sciences, 70, 554–564; Gido, K.B, Propst, D.L., Whitney, J.E., Hedden, S.C., Turner, T.F. & Pilger, T.J. (2019) Pockets of resistance: Response of arid-land fish communities to climate, hydrology, and wildfire. Freshwater Biology, 64, 761–777.
11	USA	6	Kesner, B.R. & Marsh, P.C. (2010) Central Arizona project fish monitoring: Final report. Analysis of fish population monitoring data for selected waters of the Gila River Basin, Arizona, for the five year period 2005-2009. Contract No. R09PD32013. Submitted to U.S. Bureau of Reclamation. Tempe, Arizona: Marsh and Associates, LLC. 50 pages.
18	USA	5	Pyron, M., Vaughn, C.C., Winston, M.R. & Pigg J. (1998) Fish assemblage structure from 20 years of collections in the Kiamichi River, Oklahoma. Southwestern Naturalist, 43, 336–343.
38	USA	4	Bêche, L.A., Connors, P.G., Resh, V.H. & Merenlender, A.M. (2009) Resilience of fishes and invertebrates to prolonged drought in two California streams. Ecography, 32, 778–788. The Resh Lab (2019) Hunting Creek and Knoxville Creek long-term data. Available at: https://nature.berkeley.edu/reshlab/data/HCKC_fish.xls [Accessed 05/19/2019].;
40	USA	3	U.S. Fish and Wildlife Service (2017) San Juan River basin recovery implementation program. Albuquerque, NM, 87113, USA. Data are available at https://streamsystem.org/ [Accessed on 25/05/2023].
9	USA	2	Whitney, J.E., Gido, K.E., Martin, E.C. & Hase K.J. (2016) The first to arrive and the last to leave: colonisation and extinction dynamics of common and rare fishes in intermittent prairie streams. Freshwater Biology, 61, 1321–1334.
22	USA	2	Matthews, W.J. & Marsh-Matthews, E. (2017) Data from: Stream fish community dynamics: a critical synthesis, Dryad, Dataset, doi: 10.5061/dryad.2435k.

(Continued on Next Page...)

Supplementary Table 1: **References of the dataset included in the study**, arranged by sampling size. Numeric Source ID corresponds to the ID from the RivFishTIME database (Comte et al., 2021a). N: number of sites. *(continued)*

Source ID	Country	N	Reference
23	AUS	142	Murray-Darling Basin Authority (2018) Murray-Darling Basin Fish and Macroinvertebrate Survey. Available at https://data.gov.au/data/dataset/7826d7c9-bcc5-48c0-832a-66aaedfe7b0f [Accessed on 10/15/2019].
6	AUS	117	Bunn, S.E., Abal, E.G., Smith, M.J., Choy, S.C., Fellows, C.S., Harch, B.D., Kennard, M.J. & Sheldon, F. (2010) Integration of science and monitoring of river ecosystem health to guide investments in catchment protection and rehabilitation. Freshwater Biology, 55, 223–240.
7	FIN	118	Finnish electrofishing register Hertta (2019) Available at https://wwwp2.ymparisto.fi/koekalastus_sahko/ [Accessed on 04/11/2023].
42	FIN	8	Sers, B. (2013) Swedish Electrofishing RegiSter – SERS. Swedish University of Agricultural Sciences (SLU), Department of Aquatic Resources. Available at http://www.slu.se/elfiskeregistret [Accessed on 11/18/2019].
39	CAN	97	Toronto and Region Conservation Authority (TRCA) (2018) Watershed Fish Community. Available at https://data.trca.ca/dataset/2018-watershed-fish-community [Accessed on 09/18/2019].
RAMP	CAN	13	Regional Aquatics Monitoring Program (2018). Available at http://www.ramp-alberta.org/RAMP.aspx
8	CAN	3	Sigouin, D. (2017) Fish Communities – Forillon, dataset fe2441a6-8ae4-4884-b181-cd7ec53bd842. Available at https://open.canada.ca/data/en/dataset/fe2441a6-8ae4-4884-b181-cd7ec53bd842 [Accessed on 05/07/2019].
34	ESP	100	Agencia Vasca del Agua (2019) UBEGI. Información sobre el estado de las masas de agua de la CAPV. Available at http://www.uragentzia.euskadi.eus/informacion/ubegi/u81-0003341/es/ [Accessed 10/10/2019].
43	ESP	10	Benejam, L., Angermeier, P.L., Munné, A. & García-Berthou, E. (2010) Assessing effects of water abstraction on fish assemblages in Mediterranean streams. Freshwater Biology 55, 628–642. Merciai, R., Molons-Sierra, C., Sabater, S. & García-Berthou, E. (2017) Water abstraction affects abundance, size-structure and growth of two threatened cyprinid fishes. Plos One, 12, e0175932.;
32	ESP	1	Office français de la biodiversité (2019) Suivi des éléments biologiques 'POISSONS' des rivières françaises. Available at http://www.naiades.eaufrance.fr/acces-donnees#/hydrobiologie [Accessed on 07/08/2019].
14	JPN	33	Terui, A., Ishiyama, N., Urabe, H., Ono, S., Finlay, J. C. & Nakamura, F. (2018) Metapopulation stability in branching river networks. Proceedings of the National Academy of Sciences, 115, E5963–E5969.
4	HUN	32	Eros, T., Sály, P., Takács, P., Higgins, C.L., Bíró, P. & Schmera, D. (2014) Quantifying temporal variability in the metacommunity structure of stream fishes: the influence of non-native species and environmental drivers. Hydrobiologia, 722, 31–43.

(Continued on Next Page...)

Supplementary Table 1: **References of the dataset included in the study**, arranged by sampling size. Numeric Source ID corresponds to the ID from the RivFishTIME database (Comte et al., 2021a). N: number of sites. *(continued)*

Source ID	Country	N	Reference
46	BEL	15	Van Thuyne, G., Breine, J., Verreycken, H., De Boeck, T., Brosens, D. & Desmet, P. (2013) VIS - Fishes in inland waters in Flanders, Belgium. Research Institute for Nature and Forest (INBO). Dataset/Occurrence. Available at https://www.gbif.org/dataset/823dc56e-f987-495c-98bf-43318719e30f [Accessed on 04/11/2017], doi:10.15468/gzyxyd. Brosens, D., Breine, J., Van Thuyne, G., Belpaire, C., Desmet, P. & Verreycken, H. (2015) VIS – A database on the distribution of fishes in inland and estuarine waters in Flanders, Belgium. ZooKeys, 475, 119-145.;
32	BEL	3	Office français de la biodiversité (2019) Suivi des éléments biologiques 'POISSONS' des rivières françaises. Available at http://www.naiades.eaufrance.fr/acces-donnees#/hydrobiologie [Accessed on 07/08/2019].
42	NOR	4	Sers, B. (2013) Swedish Electrofishing RegiSter – SERS. Swedish University of Agricultural Sciences (SLU), Department of Aquatic Resources. Available at http://www.slu.se/elfiskeregistret [Accessed on 11/18/2019].
35	BRA	3	Ortega, J.C.G., Dias, R.M., Petry, A.C., Oliveira, E.F. & Agostinho, A.A. (2015) Spatio-temporal organization patterns in the fish assemblages of a Neotropical floodplain. Hydrobiologia, 745, 31–41.
17	CIV	2	Levêque, C., Hougard, J.M., Resh, V., Statzner, B. & Yaméogo, L. (2003) Freshwater ecology and biodiversity in the tropics: what did we learn from 30 years of onchocerciasis control and the associated biomonitoring of West African rivers? Hydrobiologia, 500, 23–49.
31	BWA	1	Mosie, I. & Makati, K. (2018) Long term time-series data on fish monitoring by Okavango Research Institute, Botswana. Version 1.2. Okavango Research Institute. Available at https://www.gbif.org/dataset/77929c0a-7506-4b2d-a49d-10fc3312d50d [Accessed on 05/11/2019], doi: 10.15468/4vvwzc.

Supplementary Table 2: **Site location, protocol and abundance unit across sites, and data source for native species status**.

Method	Value	N	Percent
Site location (Country, Realm)	(GBR, Palearctic)	1282	28.64%
	(FRA, Palearctic)	935	20.89%
	(SWE, Palearctic)	819	18.30%
	(USA, Nearctic)	784	17.52%
	(AUS, Australasia)	213	4.76%
	(FIN, Palearctic)	126	2.82%
	(CAN, Nearctic)	113	2.52%
	(ESP, Palearctic)	111	2.48%
	(JPN, Palearctic)	33	0.74%
	(HUN, Palearctic)	32	0.71%
	(BEL, Palearctic)	18	0.40%
	(NOR, Palearctic)	4	0.09%
	(BRA, Neotropics)	3	0.07%
	(CIV, Afrotropics)	2	0.04%
	(BWA, Afrotropics)	1	0.02%
Protocol	Electrofishing	4376	97.77%
	Seining / Netting	76	1.70%
	Rotenone Lockchamber	20	0.45%
	Trapping	2	0.04%
	Trawling	2	0.04%
Abundance unit	Individual numbers per 100m2	2349	52.48%
	Count	2083	46.54%
	Catch Per Unit Effort (CPUE)	40	0.89%
	Leslie index	4	0.09%
Source for native species status	Tedesco et al. (2017) (basin scale)	325997	94.34%
	Fishbase (country scale)	19105	5.53%
	National atlases, fishbase (country and regional scale)	285	0.08%
	NAS database (state scale, USA)	157	0.05%

Supplementary Table 3: **Summary descriptors of response and predictor variables used in the full hierarchical Bayesian model** (Fig. 3, main text). For the stream longitudinal position, we display the summary descriptors of the variables used to contruct the composite variable (See Methods, main text).

Type	Variable	N	Median (Q1, Q3)	(Min, Max)
	Abundance (total) (Ind.100m2)	24315	28.00 (10.81,62.60)	(0.01,51800.00)
	Abundance (total) (Count)	22060	289.00 (128.00,602.00)	(1.00,109088.00)
	Abundance (total) (CPUE)	493	764.33 (124.00,1976.00)	(0.01,16765.50)
	Abundance (total) (Leslie_index)	64	189.05 (69.25,339.50)	(1.00,888.00)
Response	Richness	46932	4.43 (2.00,8.50)	(1.00,85.71)
	Non-native abundance	46932	0.00 (0.00,0.01)	(0.00,1.00)
	Non-native richness	46932	0.00 (0.00,0.11)	(0.00,1.00)
	Dissimilarity	46932	0.26 (0.03,0.60)	(0.00,1.00)
	Turnover	46932	0.00 (0.00,0.33)	(0.00,1.00)
	Time (year nb since first sampling)	46932	9.00 (4.00,15.00)	(0.00,59.00)
Predictor	Past pressures	46932	16.80 (8.80,26.42)	(0.20,45.60)
	Recent pressures	46932	0.98 (0.84,1.02)	(0.23,18.09)
	Annual average discharge (m3/s)	4476	1.72 (0.61,6.79)	(0.01,7830.69)
	Distance from source (km)	4476	26.60 (13.80,57.50)	(2.70,2798.30)
Stream long. position	Average elevation (m)	4476	174.00 (82.00,307.00)	(-1.00,2531.00)
	Strahler order	4476	2.00 (1.00,3.00)	(1.00,9.00)
	Average slope (degree)	4476	30.00 (16.00,51.00)	(0.00,362.00)

Supplementary Table 4: **Watanabe-Akaike Information Criterion (WAIC) of the statistical models fitting temporal trends** with either the number of years (#) since the start of sampling or log-transformed number of years plus one (See Methods). Most community metrics are best modelled with the log-transformed values.

	WAIC			
Response	Log (Year # + 1)	Year #	Min WAIC	WAIC difference (to Year nb)
Abundance (total)	8008	7623	Year #	5.1%
Richness	5427	5400	Year #	0.5%
Non-native abundance	13125	14300	Log (Year # + 1)	-8.2%
Non-native richness	13396	14459	Log (Year # + 1)	-7.4%
Dissimilarity	4559	5606	Log (Year # + 1)	-18.7%
Turnover	3956	4750	Log (Year # + 1)	-16.7%
Jaccard dissimilarity	4444	5300	Log (Year # + 1)	-16.2%
Nestedness	3986	4794	Log (Year # + 1)	-16.9%

Supplementary Table 5: **Variance inflation factors (VIF) of the ecological drivers included in the hierarchical Bayesian model** (Fig. 3, main text). SE factor: inflation of the standard error of the slope coefficients predicted by the multicollinearity of the variables. The VIF values were all close to 1, indicating absence of multicollinearity.

Predictive variables	VIF	SE factor
Time	1.00	1.00
Long. stream position	1.02	1.01
Recent pressures	1.01	1.01
Past pressures	1.02	1.01
